# Patients With Autoimmune Thyroiditis Present Similar Immunological Response to COVID-19 BNT162b2 mRNA Vaccine With Healthy Subjects, While Vaccination May Affect Thyroid Function: A Clinical Study

**DOI:** 10.3389/fendo.2022.840668

**Published:** 2022-02-22

**Authors:** Stavroula A. Paschou, Vangelis Karalis, Theodora Psaltopoulou, Vasiliki Vasileiou, Ioanna Charitaki, Tina Bagratuni, Vassiliki Ktena, Fotini Papandroulaki, Sentiljana Gumeni, Georgia N. Kassi, Ioannis P. Trougakos, Evangelos Terpos, Meletios A. Dimopoulos

**Affiliations:** ^1^ Endocrine Unit and Diabetes Center, Department of Clinical Therapeutics, Alexandra Hospital, School of Medicine, National and Kapodistrian University of Athens, Athens, Greece; ^2^ Faculty of Pharmacy, School of Health Sciences, National and Kapodistrian University of Athens, Athens, Greece; ^3^ Department of Clinical Therapeutics, Alexandra Hospital, School of Medicine, National and Kapodistrian University of Athens, Athens, Greece; ^4^ Department of Endocrinology, Alexandra Hospital, Athens, Greece; ^5^ Department of Cell Biology and Biophysics, Faculty of Biology, National and Kapodistrian University of Athens, Athens, Greece

**Keywords:** autoimmune thyroiditis, vaccination, COVID-19, immune response, SARS−CoV−2

## Abstract

**Background:**

This is the first study, that aimed: a) to compare immune response, namely the kinetics of neutralizing antibodies (Nabs), after vaccination with BNT162b2 mRNA vaccine (Comirnaty, Pfizer/BioNTech) between patients with autoimmune thyroiditis and controls, and b) to investigate changes in thyroid function in healthy subjects with no history of thyroid dysfunction before and after vaccination with BNT162b2 mRNA vaccine (Comirnaty, Pfizer/BioNTech).

**Methods:**

The entire study consisted of two sub-studies. In the first sub-study, NAbs levels after BNT162b2 mRNA vaccination were compared between 56 patients with autoimmune thyroiditis and 56 age and gender-matched healthy controls from the day of the first dose until a period of up to three months after the second dose. In the second sub-study, thyroid hormones (T3, T4, TSH) and thyroid auto-antibodies levels (anti-TG, anti-TPO) of 72 healthy subjects with no history of thyroid disease were examined before (D1) and one month after completion of the second dose (D50).

**Results:**

Among patients with autoimmune thyroiditis, the median neutralizing inhibition on D22, immediately before second dose, was 62.5%. One month later (D50), values increased to 96.7%, while three months after the second dose NAbs titers remained almost the same (94.5%). In the healthy group, median NAbs levels at D22 were 53.6%. On D50 the median inhibition values increased to 95.1%, while after three months they were 89.2%. The statistical analysis did not show significant differences between two groups (p-values 0.164, 0.390, 0.105 for D22, D50 and three months). Regarding changes in thyroid function, the mean value for T4 before vaccination was 89.797 nmol/L and one month after the second dose was 89.11 nmol/L (p-value=0.649). On D1 the mean T3 value was 1.464 nmol/L, which dropped to 1.389 nmol/L on D50 (p-value = 0.004). For TSH, mean levels were 2.064 mIU/ml on D1 and fell to 1.840 mIU/ml one month after the second dose (p-value=0.037). Despite decrease, all thyroid hormone levels remained within the normal range. No changes were found for anti-TPO or anti-TG.

**Conclusions:**

This study provided evidence that patients with autoimmune thyroiditis present similar immunological response to COVID-19 BNT162b2 mRNA vaccine (Comirnaty, Pfizer/BioNTech) with healthy subjects, while vaccination may affect thyroid function.

## Introduction

Thyroid disorders are very common and affect more than 10% of the adult population in total (1), while the prevalence of undiagnosed thyroid dysfunction is very high too (1, 2). Autoimmune thyroiditis or Hashimoto’s thyroiditis, also known as chronic lymphocytic thyroiditis, is a specific autoimmune disease in which the thyroid gland is gradually destroyed. It is characterized by the presence of thyroid autoantibodies, such as against thyroid peroxidase (anti-TPO) or thyroglobulin (anti-TG). Autoimmune thyroiditis is indeed the most usual thyroid problem nowadays, leading often to subclinical or clinical hypothyroidism (1, 3).

Since the coronavirus disease 2019 (COVID-19) pandemic outbreak, interesting data have been published on the possible thyroid complications of COVID-19 (4–6), including mainly decrease in triiodothyronine (T3) and thyroid stimulating hormone (TSH) levels, as well as subacute thyroiditis (7–12). During this challenging period, physicians strived not only to treat new cases but also to support patients with chronic disorders. Vaccination for SARS−CoV−2 is the most powerful and promising tool against the pandemic. Several questions have been raised about the safety and efficacy of vaccines in patients with existing medical problems, including those with autoimmune thyroiditis.

Scientific endocrine societies have reported early that COVID-19 vaccines are safe and recommended that all endocrine patients should be vaccinated, including those with autoimmune thyroiditis (13, 14). However, no data exist so far regarding the immunological response to COVID-19 vaccination of these patients. On the other hand, several cases of thyroid dysfunction following SARS-CoV-2 vaccination, with the majority of vaccines available, have also been described (15–29). The main complication reported is subacute thyroiditis (15–26), while there are also few cases of Graves’ disease (27–29). Taking into consideration the vaccination of millions of people worldwide, these cases represent very rare conditions. On the other hand, thyroid dysfunction may be under-reported and no robust data deriving from a properly performed study exist so far regarding the possible changes of thyroid function after vaccination against SARS-CoV-2.

This study had two aims: a) to compare immune response, namely the kinetics of neutralizing antibodies (Nabs), against SARS-CoV-2 after vaccination with BNT162b2 mRNA vaccine (Comirnaty, Pfizer/BioNTech) between patients with autoimmune thyroiditis and healthy subjects, and b) to investigate any changes in thyroid function in healthy subjects with no history of thyroid dysfunction before and after vaccination with BNT162b2 mRNA vaccine (Comirnaty, Pfizer/BioNTech).

## Patients and Methods

### Clinical Setting

The study was carried out at the Department of Clinical Therapeutics, Alexandra Hospital, School of Medicine, National and Kapodistrian University of Athens after approval from the relevant Ethical Committee. The entire clinical part followed the Helsinki Declaration and the International Conference on Harmonization for Good Clinical Practice. All subjects provided informed consent prior to participation in the study. The primary inclusion criteria for this trial were vaccination with the BNT162b2 mRNA vaccine (Comirnaty, Pfizer/BioNTech), being over the age of 18, and being able to sign informed permission. The key exclusion criteria were active malignant disease, use of immunosuppressive medications, and end-stage renal disease. Subject information was kept private in compliance with the General Data Protection Regulation. All names were kept anonymous. To prevent the patient from being identified, names were removed immediately after collecting and replaced with a random number.

The entire study was divided into two sub-studies. The first sub-study followed participants from the day of the first vaccination until a period of up to three months after the second dose. Specifically, NAbs levels after vaccination were compared between 56 patients with known autoimmune thyroiditis (positive anti-TPO or/and anti-TG) but otherwise healthy and 56 healthy controls with no history of thyroid disease, who were age and gender-matched with patients. In the second sub-study, thyroid hormones [total triiodothyronine (T3), total thyroxine (T4), thyroid stimulating hormone (TSH)] and thyroid auto-antibodies levels (anti-TG, anti-TPO) of 72 healthy subjects with no thyroid disease or such history were examined before (D1) and one month after completion of the second dose (D50).

### Neutralizing Antibodies Measurement

Blood was collected on the following days: D1 before the first vaccine, D8, D22 (the day of the second immunization right before the injection), D50, and 3 months afterwards. Serum was extracted and stored at -80°C within 4 hours of blood collection. The FDA-approved cPassTM SARS-CoV2 Nabs Detection Kit was used to measure SARS-CoV-2 neutralizing antibodies (GenScript, Piscataway, NJ, USA).

#### Thyroid Hormones Assays

Blood was collected on the following days: D1 before the first vaccine and one month after completion of the second dose (D50). T3, T4, TSH, anti-TG and anti-TPO were measured with electrochemiluminescence during the same day for all blood samples (Roche Cobas for anti-TPO, Siemens Immulite for the rest).

### Statistical Analysis

The statistical analysis began with descriptive criteria and estimation of dispersion metrics. A normality test was performed prior to statistical comparisons between two or more groups. To determine the normality of the data distribution, the Shapiro-Wilk test was used. If the nominal normality hypothesis is denied, the data is regarded to not follow the normal distribution. In circumstances where the data was determined to have a normal distribution, parametric approaches, specifically the independent t-test, were used to compare two independent groups (e.g., healthy subjects vs. thyroid patients). When comparing two groups, such as before and after immunization, the paired t-test was used. Non-parametric methods were utilized for future statistical analysis when the data distribution departed from normality. The Mann Whitney U test was employed for two independent group comparisons, such as determining the gender effect. The Wilcoxon signed-rank test was employed for pairwise group comparisons, such as neutralizing antibody levels between two occasions. The effect of age and gender on the difference in immune response between thyroid and healthy persons was also evaluated using general linear models with NAbs levels as the response variable and either analysis of variance (ANOVA) or Kruskal-Wallis. For comparisons of nominal characteristics, such as the correlation between the binary variable anti-TG or anti-TPO and vaccination, chi-square analysis was performed. Body mass index (BMI) was calculated for each subject based on the height and weight measurements. According to the BMI estimates, each participant was classified into one of the BMI groups, namely: underweight (BMI < 18.5), normal weight (BMI: 18.5 - 24.9), overweight (BMI: 25 - 29.9), obese (BMI ≥ 30).

To better understand and illustrate the changes in hormone levels before and after vaccination, their differences were also estimated by subtracting the values before vaccination (i.e., at D1) from those at D50. Therefore, new variables were created: Diff_T3, Diff_T4, Diff_TSH, Diff_anti-TPO and Diff_anti-TG. In addition, the calculation of hormone differences allowed further investigation of the relationship between the difference measures and the levels of T3, T4, TSH, anti-TPO, anti- TG. This was to investigate whether there was some kind of relationship between the hormone levels and the degree of their decline. The bivariate Pearson correlation coefficient was estimated to express the degree of their relationship.

In this study, it was also investigated whether or not the decrease in hormone levels could be associated with the adverse events after vaccination. Adverse events after vaccination were related to local effects (e.g., pain or swelling at the vaccination site, restriction of hand movement), fatigue, arthralgias/myalgias/chills/fever, headache, dizziness/sleepiness, allergies (such as itching, runny nose, redness), anaphylaxis, and others that did not fall into any of the above categories. The association between adverse events and hormone levels was examined using parametric or nonparametric comparative methods, as described above. Due to the limited sample size, the study could only be conducted with the occurrence of adverse events (i.e., yes/no) and no further analysis could be done regarding the type of adverse event, frequency, etc.

In all cases in this study, the type I error (significance level) was set at 5% and a result was considered significant if the estimated p-value (p) was less than the significance level. All statistical analysis was performed in Python v.3.9.2.

## Results

### Baseline Characteristics

The entire study consisted of two sub-studies.

In the first study, NAbs levels after a specific vaccination were compared between 56 patients with autoimmune thyroiditis and 56 healthy controls (**Table 1**).

**Table 1:** Characteristics of patients with autoimmune thyroiditis and healthy controls participating in the first sub-study.

1^st^ Sub-study	Patients with autoimmune thyroiditis	Healthy controls
Sample size	56	56
Men	5 (8.9%)	6 (10.7%)
Women	51 (91.1%)	50 (89.3%)
Age (median) years	52.0	51.0
BMI (median) kg/m^2^	24.3	23.9
Underweight (n, %)	1 (1.8%)	2 (3.6%)
Normal weight (n, %)	29 (51.8%)	31 (55.4%)
Overweight (n, %)	17 (30.4%)	16 (28.6%)
Obese (n, %)	9 (16.1%)	7 (12.5%)

n, number of subjects; BMI, body mass index. Values in parentheses refer to percentages.

Statistical analyses were performed to compare the demographics of the two groups. Separate normality tests for each characteristic and group showed that age followed a normal distribution (p-value = 0.598 for patients, p-value = 0.708 for controls), but BMI did not (p-value = 0.004 for patients, p-value = 0.023 for controls). The independent t-test for age showed no statistically significant difference (p = 0.323) and for BMI the Mann-Whitney test also showed no difference (p = 0.101).

The majority of the subjects were women, namely 91.1% in the thyroid group and 89.3% in the healthy group. The median age of the two groups was 52.0 years (thyroid patients) and 51.0 years (healthy controls). The distribution of BMI values was also similar in both groups. About half of the subjects were of normal weight, fewer were overweight, and only a few belonged to either the underweight or obese group.

In the second study, hormone levels of 72 healthy subjects with no history of thyroid disease were examined before and after vaccination (**Table 2**). The median age was 45 years and almost three quarters of the participants were women, while half of all 72 subjects were of normal weight.

**Table 2:** Characteristics of healthy subjects in whom thyroid levels were measured before (D1) and one month after the second vaccination (D50).

2^nd^ Sub-study	
Sample size	72
Men	19 (26.4%)
Women	53 (73.6%)
Age (median) years	45.0
BMI (median) kg/m^2^	24.5
Underweight (n, %)	5 (6.9%)
Normal weight (n, %)	36 (50%)
Overweight (n, %)	25 (34.7%)
Obese (n, %)	6 (8.3%)

n, number of subjects; BMI, body mass index. Values in parentheses refer to percentages.

### Neutralizing Antibodies Titers: Thyroid Patients vs. Healthy Controls

**Figure 1:**
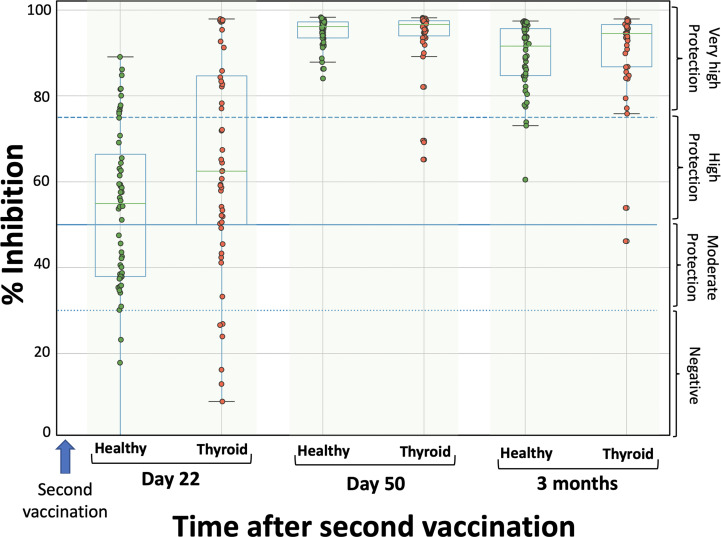
Inhibition (%) of SARS-CoV-2 binding to the human host receptor angiotensin converting enzyme in patients with autoimmune thyroiditis (red) and healthy controls (green) on the day of the second vaccination (D22), one month later (D50) and three months after the second vaccination. The boxplot boundaries show the distribution’s quartiles, whereas the superimposed dots represent individual levels of Nabs inhibition. The dashed lines represent the boundary levels of inhibition, which are 30%, 50%, and 75%.

**Figure 1** shows the percentage inhibition of NAbs on the day of the second dose, one month later (i.e., D50 after the start of the study), and three months after the second dose. Among patients with autoimmune thyroiditis, the median neutralizing inhibition on D22, immediately before vaccination, was 62.5%, while 6 individuals (8.33%) had inhibition values below the threshold of 30%. There were also 18 individuals (25.0%) with values corresponding to very high protection (NAbs titers above 75%). One month later (day 50), median inhibition values had increased to 96.7%, while three months after the second dose, NAbs titers had remained almost the same (94.5%). In the group of healthy subjects, median NAbs levels at day 22 were 53.6% and only three of them (5.1%) had levels below the lower limit of 30%. On day 50, the median inhibition values increased to 95.1%, while after three months they were 89.2% (**Figure 1**).

Normality test showed that NAbs values were not normally distributed (for all three cases p-values < 0.05), for this reason Mann-Whitney test was used for the comparisons between thyroid and healthy control groups. The statistical comparison did not show significant differences between the two groups on any day; the p-values were 0.164, 0.390 and 0.105 for D22, D50 and three months, respectively. Thus, it can be considered that there is no difference in the immune response after vaccination between patients with autoimmune thyroiditis and healthy subjects. The kinetics of NAbs follow the same pattern, increasing at D50 and slowly decreasing from that point on.

The possible influence of other factors such as age and gender has also been studied. It was found that gender had no significant influence on any of the measurement days (D22, D50, three months). The p-values of gender contribution in Kruskal-Wallis model were 0.237, 0.434 and 0.488 for D22, D50 and three months respectively. It is possible that the limited number of males (26.4% and 11.9% for the thyroid and control groups, respectively) hindered the detection of such differences.

Age was found to play a significant role in neutralizing antibody values. At D22 (i.e., three weeks after the first dose), age statistically significantly (p = 0.002 < 0.05) affected SARS Cov-2 inhibition by neutralizing antibodies and contributed with a negative sign (-0.480) to the model, suggesting that with increasing age, NAbs levels become lower. Similar results were observed at D50, where age again made a significant contribution (p = 0.043) with a negative sign (-0.169). However, three months after the second dose, no significant contribution of age was observed, suggesting that it does not play a role in the decrease of NAbs. This feature is also confirmed by the fact that age contributes more to the expression of NAbs values at D22 than at D50. In fact, the model constant (in absolute terms) is larger at D22 (i.e., -0.480) than the estimate at D50 (-0.169). This finding suggests that the influence of age gradually decreases as we move away from the day of vaccination.

### Thyroid Function Before vs. After Vaccination

The second purpose of the study was to evaluate thyroid function in a healthy group of individuals before and after vaccination. We studied in purpose healthy individuals, in order to avoid biases derived from autoimmunity or levothyroxine treatment. In this case, thyroid hormones (T3, T4, TSH) and thyroid auto-antibodies (anti-TG, anti-TPO) were measured on D1 (immediately before vaccination) and on D50 (i.e., one month after the second administration of the vaccine). The normal ranges are: T3 0.84-2.6 nmol/L, T4 58-161 nmol/L, TSH 0.4-4 mIU/ml, anti-TPO <34 IU/ml, anti-TG <40 IU/ml.

**Figure 2:**
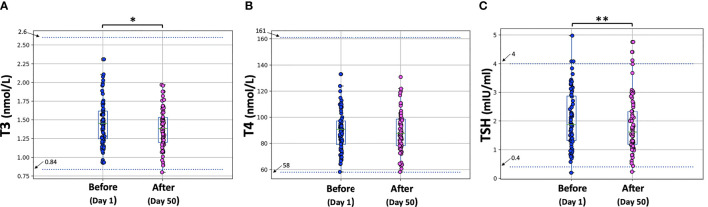
T3, T4, and TSH levels before the first vaccine dose (D1) and one month after the second vaccination (D50). Asterisks indicate statistically significant differences (p-value < 0.05) between the two comparison groups (pre- and post-vaccination) for T3 (*) and TSH (**). The boxplot boundaries show the distribution’s quartiles, while the superimposed dots represent the individual values of Nabs inhibition.

The hormone levels are shown in **Figure 2**.

Paired tests were performed to compare T3, T4, and TSH levels between D1 (before vaccination) and D50 (one month after the second dose). All three characteristics (T3, T4, TSH) were normally distributed at both days (D1, D50) according to the Shapiro-Wilk test. The p-values at D1 were 0.699, 0.756 and 0.551 for T3, T4 and TSH, respectively. For D50, the corresponding p-values were: 0.959, 0.849, 0.125. Therefore, the parametric paired t-test was used for the comparisons and the “mean” estimates were used instead of the “median” values to describe the profiles.

On D1, the mean T3 value was 1.464 nmol/L, which dropped to 1.389 nmol/L on D50. This decrease proved to be statistically significant (p-value = 0.004 < 0.05). For T4, the mean value for all subjects before vaccination was 89.797 nmol/L and one month after the second dose the mean value was 89.11 nmol/L, which is not a significant difference (p-value = 0.649 > 0.05). For TSH, mean levels were 2.064 mIU/ml on D1 and fell to 1.840 mIU/ml one month after the second dose. This decrease was also found to be significant at the 5% level (p-value = 0.037). Despite the decrease in T3 and TSH, all thyroid hormone levels remained within the normal range.

**Figure 3:**
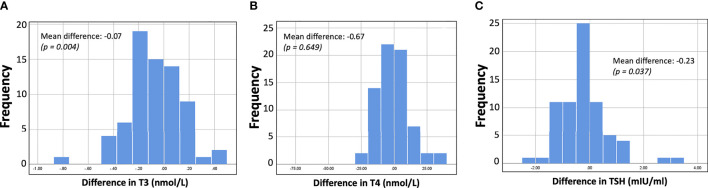
Histograms showing the distribution of differences (i.e., hormone levels after minus before vaccination). For all three hormones (T3, T4, TSH) the values decrease after vaccination, but for T3 and TSH the difference was considered significant at the 5% level.

To better examine and present the decrease in hormone levels before and after vaccination, the differences between subjects (for T3, T4, and TSH) were further estimated. These individual differences are shown graphically in **Figure 3**. **Figure 3** shows that the mean intrasubject difference is negative in all cases, i.e., all three types of hormone levels decrease after vaccination. However, this decrease was statistically significant only for T3 and TSH. In all subplots of **Figure 3**, normal distribution of the differences can be observed. Indeed, normality tests with the Shapiro-Wilk criterion resulted in p-values of 0.951, 0.167, 0.200 for the differences in T3, T4, and TSH, respectively.

By incorporating the concept of differences in hormone levels, the analysis could continue with an examination of possible relationships between individual “differences” in T3, T4, and TSH and hormone levels per se (i.e., blood T3, T4, and TSH levels). This was done to examine if there was some sort of relationship between hormone levels and degree of decline. The bivariate Pearson correlation coefficient was estimated for all pairs, but no linear correlation was found in any of them. The highest correlation (Pearson correlation coefficient equal to 0.237) was observed for the relationship between Diff_T3 and T3 values on D22. However, even in this case the correlation was not significant.

The levels of anti-TPO antibodies before and after vaccination were also assessed by pairwise comparisons. However, the difference was found to be not statistically significant (p-value = 0.722). Anti-TG responses were also examined for their association with vaccination. Chi-square analysis assessed the association between anti-TG and vaccination, but no significant changes were found (p = 0.989).

We also examined whether or not the decrease in hormone levels could be associated with the occurrence of adverse events due to vaccination. In general, no statistically significant association (p-values > 0.05) was found between the decrease in hormones and occurrence of an adverse event.

## Discussion

No differences in the immunological response after vaccination with BNT162b2 mRNA vaccine (Comirnaty, Pfizer/BioNTech) between patients with autoimmune thyroiditis and healthy subjects, specifically on the day of the second dose (D22 from the start of the study), one month later (D50), and three months after the second dose, were observed. Among patients with autoimmune thyroiditis, the median neutralizing inhibition on D22 and immediately before second dose was 62.5%, one month later (D50) increased to 96.7%, while three months after the second vaccination NAbs titers had remained almost the same (94.5%). The statistical comparison did not show significant differences between patients with autoimmune thyroiditis and healthy controls on any day. The kinetics of NAbs follow the same pattern, increasing at D50 and slowly decreasing from that point on. To our knowledge this is the first study that investigated the immunological response of patients with autoimmune thyroiditis against any COVID-19 vaccine. These findings are of great importance, as Hashimoto’s thyroiditis is a very usual clinical problem worldwide nowadays, affecting around 10% of the adult population (1, 2).

Patients and controls were age and gender matched; however, we investigated the impact of these parameters in immunological response in general. Gender had no significant influence on any of the measurement days, while age was found to play a significant role in NAbs values. At D22, age statistically significantly affected the response, suggesting that with increasing age, NAbs levels become lower. Similar results were observed at D50, where age again made a significant negative contribution. However, three months after the second dose, no significant contribution of age was observed, suggesting that the influence of age gradually decreases as we move away from the day of vaccination. This is in accordance with previous findings that indicate a negative effect of age on immunological response (30).

Another finding of this study was the decrease of T3 and TSH levels after vaccination with BNT162b2 mRNA vaccine. Specifically, T3 and TSH mean levels statistically significantly decreased from D1 to D50, while T4 levels remained stable. These were observed after 4 weeks since second dose and 7 weeks since first dose. We measured thyroid hormones in purpose at this time point, as thyroid axis needs few weeks for functional changes. To better examine the decrease in hormone levels before and after vaccination, the differences between subjects (for T3, T4, and TSH) were further estimated. The mean intrasubject difference was negative in all cases; this decrease was statistically significant only for T3 and TSH too, confirming the initial findings. We need to note that despite the decrease, all thyroid hormone levels remained within the normal range. No significant changes were found for anti-TPO or anti-TG auto-antibodies.

The question about possible changes in thyroid function is crucial. Several cases of thyroid dysfunction following SARS-CoV-2 vaccination, with the majority of vaccines available, have been described (15–29), including BNT162b2 mRNA vaccine (22–26). The main complication reported is subacute thyroiditis (15–26), while there are also few cases of Graves’ disease (27–29). However, no robust data deriving from a properly performed study existed so far. Decrease in T3 and TSH levels has been reported in patients affected by COVID-19 during first days (31) or weeks after the disease (5–7). The possible thyroid dysfunction after COVID-19 vaccination cannot be completely explained. One hypothesis could be the influence of systemic immune-mediated post-vaccination inflammatory response on the thyroid gland, leading to T3 reduction, and/or on pituitary gland, leading to TSH reduction and indirect effects on thyroid function. A second potential explanation could imply an underlying nonthyroidal illness syndrome or euthyroid sick syndrome, which is often caused by illness. It is characterized by normal or low serum TSH concentration and low T3 concentration, accompanied by a normal or low T4 concentrations. This is an adaptive body mechanism to recover from, critical mainly, illness (32). However, we need to note that despite the decrease, all thyroid hormone levels remained within the normal range. Moreover, when we examined whether or not the decrease in hormone levels could be associated with the occurrence of adverse events due to vaccination, no statistically significant association was found. On top, if such thyroid hormones changes remain for longer time needs further investigation.

To our knowledge, this is the first study that investigated the immunological response of patients with autoimmune thyroiditis against any COVID-19 vaccine. Moreover, this is the first research regarding the possible changes of thyroid function after vaccination against SARS-CoV-2 in the context of a clinical study. Two separate sub-studies were performed with different populations, in order to properly investigate the two aims. We studied in purpose healthy individuals in the second sub-study, in order to avoid possible biases derived from autoimmunity or levothyroxine replacement treatment. A limitation of the study is the relatively small sample size of the two sub-studies. This small sample size may lead to low statistical power and sensitivity in detecting differences in some variables being compared. Moreover, although patients and controls were age- and sex-matched, the unequal number of males and females (the vast majority were women) could potentially influence the results on the role of sex.

In conclusion, this study provided evidence that patients with autoimmune thyroiditis present similar immunological response to COVID-19 BNT162b2 mRNA vaccine (Comirnaty, Pfizer/BioNTech) with healthy subjects, while vaccination may affect thyroid function, namely decrease TSH and T3 levels.

## Data Availability Statement

The raw data supporting the conclusions of this article will be made available by the authors, without undue reservation.

## Ethics Statement

The studies involving human participants were reviewed and approved by Ethics Committee, Alexandra Hospital, National and Kapodistrian University of Athens. The patients/participants provided their written informed consent to participate in this study.

## Author Contributions

SP designed the protocol, participated in the collection of the data and wrote the manuscript. VKa performed the statistical analysis. TP revised the manuscript. VV participated in the collection of the data. IC participated in the collection of the data. TB participated in the collection of the data. VKt participated in the collection of the data. FP participated in the collection of the data. SG participated in the collection of the data. GK revised the manuscript. IT participated in the collection of the data and revised the manuscript. ET designed the protocol, participated in the collection of the data and revised the manuscript. MD designed the protocol, participated in the collection of the data and revised the manuscript. All authors approved the final version of the article.

## Conflict of Interest

The authors declare that the research was conducted in the absence of any commercial or financial relationships that could be construed as a potential conflict of interest.

## Publisher’s Note

All claims expressed in this article are solely those of the authors and do not necessarily represent those of their affiliated organizations, or those of the publisher, the editors and the reviewers. Any product that may be evaluated in this article, or claim that may be made by its manufacturer, is not guaranteed or endorsed by the publisher.
